# A non‐judgmental companion: connections to the natural environment for people affected by dementia

**DOI:** 10.1002/alz70858_101565

**Published:** 2025-12-25

**Authors:** Veronika Williams, Mary Pat Sullivan, Christina R Victor

**Affiliations:** ^1^ Nipissing University, North Bay, ON, Canada; ^2^ Brunel University, Uxbridge, England, United Kingdom

## Abstract

**Background:**

Evidence suggests that social connections are often disrupted by a dementia diagnosis. Eco‐mapping, a common practice in social work, allows the visualization of interconnected lives and permits an opportunity to explore the nature and quality of social connections, and their protective or adverse risk factors for isolation. Their use in a research context with a focus on connections to the natural environment rather than people, is less familiar. Given the strong evidence suggesting the importance of the natural environment to our well‐being, we conducted a qualitative multi‐method study to identify the nature, quality and essence of connections to the natural environment for people affected by dementia and explore these in the context of their well‐being.

**Methods:**

Care partners, and where possible their partner living with different dementia diagnoses, developed eco‐maps during virtual research interviews to illustrate their “blue‐green‐white” connections to the environment and their quality. This was followed by a series of open‐ended questions to further explore their various connections and a subjective assessment of their social and environmental health. We also conducted walking interviews with a subset of participants to further contextualize mapping data and capture the relationship between well‐being and environment. Drawing on participatory methods, we photographed aspects during the walk deemed as significant by the participant. The different data sets were analyzed using thematic analysis and integrated at the interpretation and conceptualization stage.

**Results:**

Our sample included nineteen participants, four of whom provided data as a dyad. The eco‐maps provided an effective visual representation of the complexity of fluctuating social and environmental connections, and together with the interviews and images allowed for a more comprehensive understanding on how blue‐green‐white connections affected their well‐being, and their relationship with their partner. Our findings suggest that connections to nature provided energy, which participants were able to draw on and seek strength from, allowed them to (re) connect with themselves and their partner, and nature being a non‐judgmental companion contributing to a sense of safety and calmness.

**Conclusion:**

Connection to the natural environment can be protective for families affected by dementia and its assessment can contribute to family‐centred supports.

